# Impact of a circular powered stapler on preventing anastomotic leakage in patients with left-sided colorectal cancer: a retrospective study

**DOI:** 10.1186/s12893-023-02104-5

**Published:** 2023-07-18

**Authors:** Masatsune Shibutani, Tatsunari Fukuoka, Yasuhito Iseki, Hiroaki Kasashima, Kishu Kitayama, Kiyoshi Maeda

**Affiliations:** Department of Gastroenterological Surgery, Osaka Metropolitan University Graduate School of Medicine, 1–4–3 Asahi-machi Abeno-ku, Osaka City, 545-8585 Osaka Prefecture Japan

**Keywords:** Colorectal cancer, Powered circular stapler, Double stapling technique, Anastomotic leakage

## Abstract

**Background:**

The powered circular stapler, which was developed with the aim of providing reliable and reproducible anastomosis, provides complete anastomosis, resulting in a reduced risk of anastomotic leakage. The aim of this study was to compare the incidence of anastomotic leakage between a conventional manual circular stapler (MCS) and the ECHELON CIRCULAR™ Powered Stapler (ECPS) in patients with left-sided colorectal cancer who underwent anastomosis with the double stapling technique.

**Methods:**

A total of 187 patients with left-sided colorectal cancer who underwent anastomosis with the double stapling technique with a conventional MCS or the ECPS during surgery at Osaka City University Hospital between January 2016 and July 2022 were enrolled in this study.

**Results:**

The incidence of anastomotic leakage in the ECPS group was significantly lower than that in the MCS group (4.4% versus 14.3%, p = 0.048). Furthermore, even after propensity score matching, an association was found between the use of the ECPS and a reduced incidence of anastomotic leakage.

**Conclusion:**

The ECPS has the potential to help reduce the rate of anastomotic leakage in left-sided colorectal surgery.

## Background

Due to advances in surgical techniques and improvements in surgical devices, postoperative complications in digestive surgery have decreased [1]. However, anastomotic leakage remains a serious complication in colorectal surgery and continues to be a concern for colorectal surgeons. Anastomotic leakage can worsen not only short-term outcomes, such as the rate of reoperation and duration of hospitalization but also oncological outcomes, such as the rate of recurrence and survival time [2–4]. Furthermore, anastomotic leakage has a negative impact on economic outcomes [1, 5].

Intraoperative factors, such as intestinal perfusion, tension and incomplete anastomosis, have been reported to affect the incidence of anastomotic leakage [6–8], in addition to patient background factors, such as sex, body mass index (BMI), diabetes, tumor diameter, distance from anal verge, American Society of Anesthesiologists (ASA) physical status and neoadjuvant chemoradiotherapy [9–11]. In addition to the reinforcement of anastomosis, such as additional sutures and the use of a polyglycolic acid (PGA) sheet [12, 13], we focused on a powered circular stapler, the ECHELON CIRCULAR™ Powered Stapler (ECPS) (Ethicon, Somerville, NJ, USA), which was launched in Japan in 2020, as a measure against incomplete anastomosis. This powered circular stapler was developed with the aim of providing reliable and reproducible anastomosis [14]. The use of this new device provides complete anastomosis, resulting in a reduced risk of anastomotic leakage.

The aim of this study was to compare the incidence of anastomotic leakage between a conventional manual circular stapler (MCS) and the ECPS in patients with left-sided colorectal cancer who underwent anastomosis with the double stapling technique (DST).

## Methods

### Patients

A total of 187 patients with left-sided colorectal cancer who underwent DST anastomosis with a conventional MCS, the ENDOPATH ENDOSCOPIC INTRALUMINAL STAPLER (Ethicon, Somerville, NJ, USA) or the ECPS during surgery at Osaka City University Hospital between January 2016 and July 2022 were enrolled in this study. An expert in colorectal surgery participated in all surgeries. There was no operator bias in the use of circular staplers. High ligation of the inferior mesenteric artery was performed in all cases. Indocyanine green fluorescence imaging was performed to evaluate blood perfusion in patients who underwent surgery after June 2017. Mobilization of the splenic flexure was performed when tension at the anastomotic site was noted. Reinforcement of anastomosis, such as additional sutures and/or a PGA sheet, was added according to the judgment of the surgeon.

### Definition of anastomotic leakage

Anastomotic leakage was defined as the extravasation observed on radiologic examinations. Upon clinical symptoms that suggested anastomotic leakage, such as abdominal pain, high fever, pus or fecal discharge from the pelvic drain, or leukocytosis, computed tomography (CT) was performed to confirm the presence of anastomotic leakage. The following CT findings were considered suggestive of anastomotic leakage: abscess, fluid collection, or air bubbles surrounding the anastomotic site. Water-soluble contrast enema was also performed as needed to confirm the presence of communication between the intra- and extraluminal compartments.

### Ethics statement

This retrospective study was approved by the Ethics Committee of Osaka City University (approval number: 4182) and was conducted in accordance with the Declaration of Helsinki. Written informed consent was obtained from all patients.

### Statistical analyses

All statistical analyses were performed using the SPSS software package for Windows (IBM, Chicago, IL, USA). The significance of differences according to the circular stapler that was used and the patients’ background/operative outcomes were analyzed using the chi-squared test, Fisher’s exact test, and the Mann–Whitney U test. A logistic regression model was used to evaluate the factors associated with anastomotic leakage. P values < 0.05 were considered to indicate a statistically significant difference. To reduce the impact of selection bias and potential confounding, we performed propensity score matching. The propensity scores were estimated using multivariate logistic regression models, with the groups as the dependent variable and patient characteristics and operative outcomes as covariates. Matching was performed with a one-to-one greedy nearest neighbor algorithm with a caliper of 0.2 without replacement.

## Results

The MCS was used in 119 cases (MCS group), and the ECPS was used in 68 cases (ECPS group). Associations between the type of circular stapler and the patients’ characteristics/operative outcomes are summarized in Table 1. The ECPS was significantly associated with nonsmokers, lower BMI, a higher proportion of robot-associated surgery and smaller circular staplers and tended to be associated with lower rectal cancer. Associations between the type of circular stapler and postoperative complications are summarized in Table 2. The incidence of anastomotic leakage in the ECPS group was significantly lower than that in the MCS group (4.4% versus 14.3%, p = 0.048). The reoperation rate in the ECPS group was lower than that in the MCS group (1.5% versus 2.5%, p > 0.999); however, the difference did not reach statistical significance due to the extremely small number of events. No patients with anastomotic bleeding were observed in the ECPS or MCS group. The associations between anastomotic leakage and various factors are shown in Table 3. According to a univariate analysis, anastomotic leakage showed significant associations with male sex, large tumor, absence of diverting ileostomy, long operation time, and manual circular stapler. Furthermore, even after propensity score matching using the three covariates of BMI, tumor location, and smoking, which have been reported to affect anastomotic leakage and which showed significant differences in patient backgrounds, the ECPS remained associated with a reduced incidence of anastomotic leakage.

**Table 1:** Associations between the type of circular stapler and patient characteristics/operative outcomes

	Entire cohort				Matched cohort		
Factors	The ECPS group (n = 68)	The MCS group (n = 119)	p-value		The ECPS group (n = 63)	The MCS group (n = 63)	p-value
Age (years)							
Median (range)	69(42–91)	68(41–87)	0.964		69(42–91)	68(41–87)	0.847
Sex, n							
Male	32	48			30	26	
Female	36	71	0.443		33	37	0.591
ASA physical status, n							
1, 2	54	103			50	58	
3	14	16	0.218		13	5	0.073
Diabetes mellitus							
Absent	58	93			53	50	
Present	10	26	0.254		10	13	0.645
Steroid use							
Absent	68	118			63	63	
Present	0	1	> 0.999		0	0	> 0.999
Smoking							
Absent	51	65			46	46	
Present	17	54	0.008		17	17	> 0.999
BMI (mg/m^2^)							
Median (range)	21.24 (14.84–40.88)	22.77 (15.26–32.19)	0.013		21.23 (14.84–40.88)	22.21 (15.26–32.19)	0.155
Tumor location, n							
Above the peritoneal reflection	57	110			57	57	
Below the peritoneal reflection	11	9	0.085		6	6	> 0.999
Tumor diameter (cm)							
Median (range)	3.5 (0–12.0)	4.0 (0–10.0)	0.223		3.5 (0–12.0)	4.0 (0–10.0)	0.182
Pathological stage							
0	2	3			2	1	
I	23	36			22	19	
II	20	47			19	25	
III	16	22			13	10	
IV	7	11	0.732		7	8	0.767
Neoadjuvant chemoradiotherapy, n							
Absent	67	116			62	61	
Present	1	3	> 0.999		1	2	> 0.999
Surgical approach, n							
Open	3	13			3	9	
Laparoscopic	39	97			39	48	
Robot-assisted	26	9	< 0.001		21	6	0.002
Number of stapler cartridges for rectal transection, n							
1, 2	65	117			60	62	
3	3	2	0.355		3	1	0.619
Diameter of circular stapler (mm), n							
25	51	46			47	25	
29	17	73	< 0.001		16	38	< 0.001
Additional reinforcement, n							
Absent	27	46			24	26	
Present	41	73	> 0.999		39	37	0.856
Diverting ileostomy, n							
Absent	58	110			56	58	
Present	10	9	0.136		7	5	0.763
Intraoperative blood loss (ml)							
Median (range)	20 (5-1080)	20 (5-2730)	0.597		20 (5-1080)	20 (5-2730)	0.25
Operation time (min)							
Median (range)	238 (117–1129)	247 (127–666)	0.882		228 (117–1129)	245 (130–666)	0.408

ECPS: ECHELON CIRCULAR™ Powered Stapler; MCS: manual circular stapler; ASA: American Society of Anesthesiologists; BMI: body mass index

**Table 2:** Postoperative complications

	Entire cohort				Matched cohort		
Factors	The ECPS group (n = 68)	The MCS group (n = 119)	p-value		The ECPS group (n = 63)	The MCS group (n = 63)	p-value
Anastomotic leakage, n (%)	3 (4.4%)	17 (14.3%)	0.048		2 (3.2%)	9 (14.3%)	0.054
Reoperation, n (%)	1 (1.5%)	3 (2.5%)	> 0.999		0 (0%)	3 (4.8%)	0.244
Postoperative bleeding at anastomotic site, n (%)	0 (0%)	0 (0%)	N/A		0 (0%)	0 (0%)	N/A
Mortality, n (%)	0 (0%)	0 (0%)	N/A		0 (0%)	0 (0%)	N/A

ECPS: ECHELON CIRCULAR™ Powered Stapler; MCS: Manual circular stapler; N/A: not applicable

**Table 3:** Univariate analyses of risk factors for anastomotic leakage

Factors	Hazard Ratio	95% CI	p-value
Age (≥ 75 vs. <75 years)	0.602	0.192–1.892	0.385
Sex (Male vs. Female)	7.885	1.774–35.054	0.007
ASA physical status (3 vs. 1–2)	1.893	0.632–5.675	0.254
Diabetes mellitus (Absent vs. Present)	1.462	0.494–4.326	0.492
Steroid use (Absent vs. Present)			N/A
Smoking (Absent vs. Present)	0.575	0.227–1.461	0.245
BMI (≥ 25 vs. <25 kg/m2)	1.505	0.564–4.016	0.414
Tumor location (Below the peritoneal reflection vs. Above the peritoneal reflection)	1.557	0.414–5.863	0.513
Tumor diameter (≥ 5 vs. <5 cm)	2.860	1.116–7.328	0.029
Neoadjuvant chemoradiotherapy (Present vs. Absent)	2.877	0.285–29.058	0.370
Number of stapler cartridges for rectal transection (3 vs. 1–2)	2.145	0.228–20.191	0.505
Diameter of circular stapler (29 vs. 25 mm)	1.361	0.536–1.361	0.516
Additional reinforcement (Absent vs. Present)	1.214	0.460–3.201	0.696
Diverting ileostomy (Absent vs. Present)	3.643	1.153–11.511	0.028
Intraoperative blood loss (≥ 100 vs. <100 ml)	1.296	0.402–4.179	0.664
Operation time (≥ 360 vs. <360 min)	2.792	1.020–7.642	0.046
Circular Stapler (Manual vs. Powered)	3.611	1.018–12.810	0.047

CI: confidence interval; ASA: American Society of Anesthesiologists; BMI: body mass index

## Discussion

This study demonstrated that the use of the ECPS reduces the rate of anastomotic leakage in left-sided colorectal surgery, as demonstrated in previous reports [15–17]. To our knowledge, this is the first report from Asia to evaluate the usefulness of the ECPS for the prevention of anastomotic leakage in colorectal surgery. In this study, the ECPS group had some adverse conditions (e.g., lower anastomosis) in comparison to the MCS group. Nevertheless, the outcomes in the ECPS group were better than those in the MCS group. Furthermore, even after propensity score matching, an association was found between the use of the ECPS and a reduced incidence of anastomotic leakage. These results strongly support the usefulness of the ECPS.

The introduction of the circular stapler in colorectal surgery has greatly contributed not only to shortening the operation time but also to the establishment of stable procedures that are not affected by the skill of the surgeon and to the improvement of clinical outcomes [15]. In addition to various past improvements in circular stapler technology, the ECPS was developed based on the concept of increased strength at the anastomotic site, a gentler approach to the tissue, minimization of variation in usage and standardization of performance across users [14, 18]. The details of the improvements are as follows.

First, the powered stapling system, which is operated by electricity, was introduced [17]. Since MCS requires a strong force to fire, movement at the distal tip becomes large when using MCS, especially when performed by a surgeon with small hands or insufficient grip strength [17, 19]. Therefore, MCS may cause suboptimal tension on the tissue at the anastomotic site, which may cause microtissue damage, leading to anastomotic trouble [19–21]. On the other hand, the ECPS requires less force to fire. Therefore, movement at the distal tip can be reduced, resulting in a reduction in suboptimal tissue tension at the anastomotic site and providing more stable stapler positioning and staple line formation [14, 15, 17–19].

Second, 3D stapling technology has been newly introduced. Although the staple array of the ECPS is 2 rows, the same as the MCS, the area covered by one staple of the ECPS is increased in comparison to the MCS due to the use of a 3D staple shape that compresses the tissue widely and sterically [15, 19]. As a result, the physical pressure resistance increases in comparison to the MCS. Although it is unclear whether there is a direct correlation between physical pressure resistance and the rate of anastomotic leakage, it is an undeniable fact that the use of the ECPS dramatically reduced the rate of anastomotic leakage in several previous reports [15–17]. Furthermore, it has been reported that improved staple formation contributes to the prevention of bleeding at the anastomotic site as well as anastomotic leakage [14].

Third, Gripping Surface Technology (GST), which provides gentler handling with precise compression only where it is needed, has also been introduced [15]. This improvement minimizes tissue damage, resulting in a 33% reduction in compressive force on tissue.

As described above, the ECPS has been improved in various points in comparison to the MCS. Nevertheless, the increase in selling price is set to be mild. Thus, the ECPS may account for most of the circular stapler market share in the near future.

In this study, small-diameter circular staplers were more frequently used in the ECPS group than in the MCS group. The reason for this is the recent increase in the use of smaller-sized circular staplers, based on the hypothesis that the use of circular staplers of smaller diameter may reduce the overstretching of tissue, resulting in less anastomotic trouble. However, the size of the circular stapler was not associated with the incidence of anastomotic leakage, as documented in a previous report [22].

In the current study, anastomotic leakage was observed in three patients in the ECPS group. All three patients had lower anastomosis and required a long operation time of ≥ 9 h. In addition, the tumor diameter was ≥ 7 cm in 2 of these 3 cases. Given these facts, there may have been invisible damage to the intestinal tract in these three patients who developed anastomotic leakage.

The present study is associated with several limitations. First, this was a retrospective study with a small cohort in a single center. Based on previous reports on medical statistics [23, 24], we abandoned the multivariate analysis with a Cox proportional hazards model for anastomotic leakage due to an insufficient total number of events. Instead, propensity score matching was performed to reduce the impact of selection bias and potential confounding factors. However, due to the small number of cases, the relationship between the reduced incidence of anastomotic leakage and the use of the ECPS showed a trend but did not reach statistical significance. Large prospective studies should be performed to confirm our findings. Second, additional reinforcement may have the potential to have some impact on the rate of anastomotic leakage, although there was no statistically significant correlation in this study because additional reinforcement tended to be omitted in patients with a low risk of anastomotic leakage. Third, the ECPS, which was launched in recent years, was more frequently used in robot-assisted surgery in comparison to the MCS due to historical backdrop. However, it has been reported that robot-assisted surgery and laparoscopic surgery are comparable in terms of postoperative complications [25].

## Conclusion

In conclusion, the ECPS has the potential to help reduce the rate of anastomotic leakage in left-sided colorectal surgery.

## Data Availability

The datasets used and/or analyzed during the current study are available from the corresponding author on reasonable request.

## Abbreviations

BMI: body mass index
ASA: American Society of Anesthesiologists
PGA: polyglycolic acid
ECPS: ECHELON CIRCULAR™ Powered Stapler
MCS: manual circular stapler
DST: double-stapling technique
CT: computed tomography
N/A: not applicable
